# GRB7 Plays a Vital Role in Promoting the Progression and Mediating Immune Evasion of Ovarian Cancer

**DOI:** 10.3390/ph17081043

**Published:** 2024-08-07

**Authors:** Liang Wen, Wei Hu, Sen Hou, Ce Luo, Yiteng Jin, Zexian Zeng, Zhe Zhang, Yuanguang Meng

**Affiliations:** 1 Chinese People’s Liberation Army (PLA) Medical School, Beijing 100853, China; 18392181763@163.com; 2 Department of Emergency, The Fifth Medical Center of Chinese PLA Hospital, Beijing 100039, China; huwei301yy@163.com; 3 Department of Gastrointestinal Surgery, Peking University People’s Hospital, Beijing 100032, China; housen95@163.com; 4 Center for Quantitative Biology, Academy for Advanced Interdisciplinary Studies, Peking University, Beijing 100091, China; ce.luo@stu.pku.edu.cn (C.L.); 2101111816@pku.edu.cn (Y.J.); zexianzeng@pku.edu.cn (Z.Z.); 5 Department of Obstetrics and Gynecology, Seventh Medical Center of Chinese PLA General Hospital, Beijing 100700, China

**Keywords:** ovarian cancer, GRB7, prognostic biomarker, immune infiltration, immunotherapy

## Abstract

Background: Despite breakthroughs in treatment, ovarian cancer (OC) remains one of the most lethal gynecological malignancies, with an increasing age-standardized mortality rate. This underscores an urgent need for novel biomarkers and therapeutic targets. Although growth factor receptor-bound protein 7 (GRB7) is implicated in cell signaling and tumorigenesis, its expression pattern and clinical implications in OC remain poorly characterized. Methods: To systematically investigate GRB7’s expression in OC, our study utilized extensive datasets from TCGA, GTEx, CCLE, and GEO. The prognostic significance of GRB7 was evaluated by means of Kaplan–Meier and Cox regression analyses. Using a correlation analysis and gene set enrichment analysis, relationships between GRB7’s expression and gene networks, immune cell infiltration and immunotherapy response were investigated. In vitro experiments were conducted to confirm GRB7’s function in the biology of OC. Results: Compared to normal tissues, OC tissues exhibited a substantial upregulation of GRB7. Reduced overall survival, disease-specific survival, and disease-free interval were all connected with high GRB7 mRNA levels. The network study demonstrated that GRB7 is involved in pathways relevant to the course of OC and has a positive connection with several key driver genes. Notably, GRB7’s expression was linked to the infiltration of M2 macrophage and altered response to immunotherapy. Data from single-cell RNA sequencing data across multiple cancer types indicated GRB7’s predominant expression in malignant cells. Moreover, OC cells with GRB7 deletion showed decreased proliferation and migration, as well as increased susceptibility to T cell-mediated cytotoxicity. Conclusion: With respect to OC, our results validated GRB7 as a viable prognostic biomarker and a promising therapeutic target, providing information about its function in tumorigenesis and immune modulation. GRB7’s preferential expression in malignant cells highlights its significance in the biology of cancer and bolsters the possibility that it could be useful in enhancing the effectiveness of immunotherapy.

## 1. Introduction

Ovarian cancer (OC) remains the most lethal gynecological malignancy. The International Agency for Research on Cancer estimated that there were more than 300,000 new cases of ovarian cancer and more than 200,000 ovarian cancer deaths in the year 2022 [1]. Between 2007 and 2017, the global age-standardized incidence and disability-adjusted life-year rates of OC increased by 2.05% and 1.34%, respectively [2]. Additionally, the age-standardized mortality rate of OC increased from 1.76 to 2.88 per 100,000 from 1990 to 2019 in China [3]. Ovarian cancer is the worst prognosis tumor among female reproductive system tumors with an age-standardized 5-year net survival of 43.4% [4], largely due to late diagnosis and frequent relapse after initial treatment. In addition, ovarian cancer responds poorly to immune therapy, and the objective response rates were only 7.4% and 9.9% in two different cohorts [5]. Therefore, we need more effective therapeutic regimens to treat ovarian cancer. However, the complexity of tumor biology, including the interplay between tumor cells and the immune microenvironment, poses a significant challenge to therapeutic advancements.

The growth factor receptor-bound protein 7 (GRB7) belongs to a family of adapter proteins implicating in various cellular processes and plays significant roles in cell signaling pathways [6]. Due to its overexpression and possible role in the initiation and spread of several malignancies, including breast and colon cancer, GRB7 has become a noteworthy molecule [7,8]. Yet, the comprehensive role of GRB7 in ovarian cancer, including its expression patterns, prognostic value, and therapeutic potential, has not been fully elucidated.

Using omics data produced at several sequencing platforms, this study methodically analyzed the expression patterns of GRB7 and examined the prognostic significance of GRB7 in OC and its relationship to patient prognosis. We conducted a thorough investigation to investigate the expression of GRB7 in OC using datasets from The Cancer Genome Atlas (TCGA), Genotype-Tissue Expression (GTEx), Cancer Cell Line Encyclopedia (CCLE), and Gene Expression Omnibus (GEO). We conduct gene network construction, survival analyses, and evaluations of GRB7’s impact on immune infiltration and immunotherapy responsiveness. Importantly, our findings revealed that GRB7 is preferentially expressed in malignant cells compared to immune cells across a variety of cancer types, suggesting that GRB7 may be a promising target for future therapeutics. To support this, we assessed the effect of GRB7 deletion on the migration, proliferation, and sensitivity of OC cells to T cell-mediated cytotoxicity using in vitro tests.

## 2. Results

### 2.1. GRB7’s Expression Is Upregulated in Ovarian Cancer Tissue

**Figure 1:**
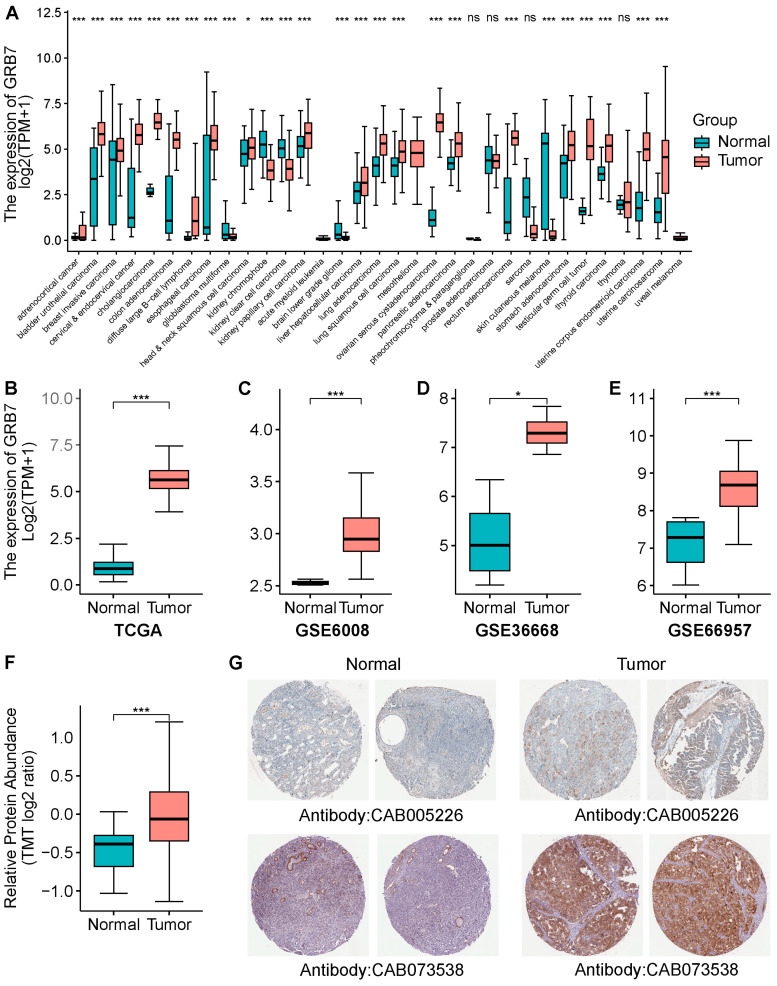
The expression of GRB7. (**A**,**B**) GRB7 mRNA levels in pan-cancer (**A**), OC (**B**), and the corresponding normal tissues in TCGA and GTEx databases. (**C**–**E**) GRB7’s expression in OC and normal tissues in GEO databases, GSE6008 (**C**), GSE36668 (**D**), and GSE66957 (**E**). (**F**) GRB7 protein levels in OC and paired adjacent normal tissues from cProCite database. (**G**) Representative results of immunochemically stained GRB7 proteins in OC and normal ovarian tissues from Human Protein Atlas. * *p* < 0.05; *** *p* < 0.001 by unpaired Student’s *t* test (**A**–**F**). ns, not significant.

Analyses of Cancer Cell Line Encyclopedia (CCLE) datasets showed that both mRNA and protein levels of GRB7 were higher in OC than in most other cancers (Figure S1A,B). Further analyses of TCGA and GTEx databases showed significant upregulation of GRB7 in various cancers compared to their matched normal controls (Figure 1A). Notably, the mRNA levels of GRB7 were significantly higher in ovarian cancer tissues than in normal tissues, as confirmed by TCGA-GTEx (Figure 1B) and GEO data (Figure 1C–E). Protein levels further validated significant upregulation in ovarian cancer tissues compared to the controls (Figure 1F), with immunohistochemical staining from the Human Protein Atlas (HPA) corroborating higher expression in OC tissue (Figure 1G and Figure S2A,B).

### 2.2. GRB7’s Expression Is Independently Associated with a Poorer Outcome and Is Valuable for Predicting OS in OC Patients

**Figure 2:**
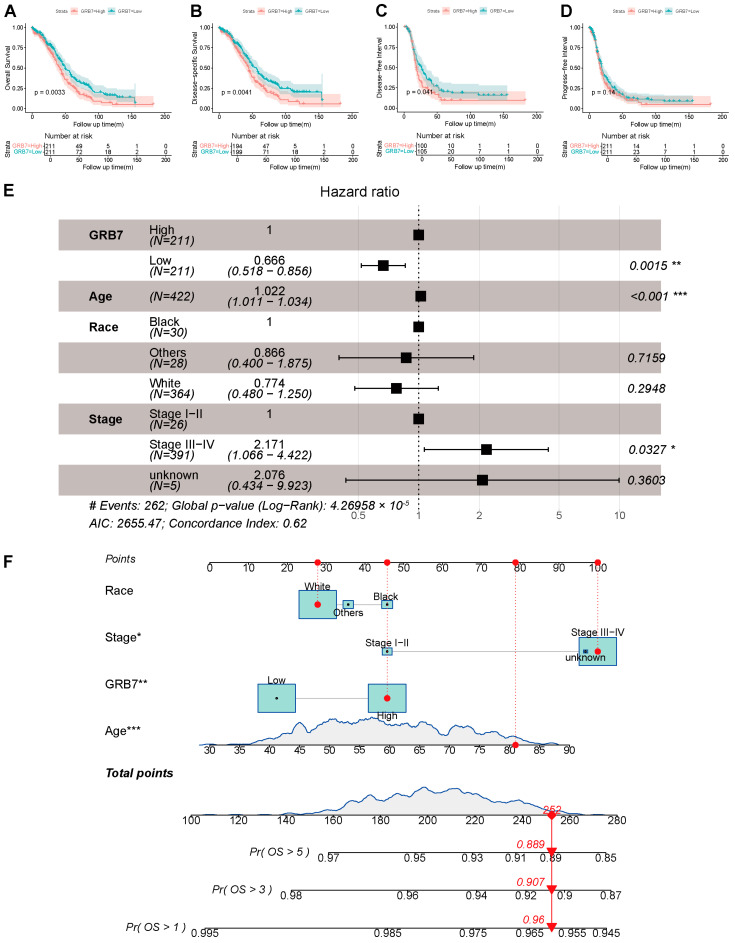
The prognosis and diagnosis value of GRB7 in OC. (**A**–**D**) OS, DSS, DFI, and PFI curves of lowly and highly expressed GRB7 in OC. (**E**) Univariate and multivariate regression analyses of GRB7 and clinicopathologic factors with OS in OC patients from TCGA. (**F**) A nomogram to predict OS probability at 1-year, 2-year, and 3-year overall survival probabilities for OC. * *p* < 0.05; ** *p* < 0.01; *** *p* < 0.001 by unpaired Student’s t test (E, F). # Events represents the number of death cases.

In OC patients, high GRB7 mRNA levels are associated with poor overall survival (OS), disease-free interval (DFI), and disease-specific survival (DSS) but not significantly with progress-free interval (PFI) based on TCGA (Figure 2A–D). To further verify the predictive power of GRB7 in ovarian cancer overall survival, we conducted univariate and multivariate regression analyses of GRB7 and clinicopathologic factors with OS in OC patients from TCGA and found that it may be an independent risk factor for ovarian cancer (Figure 2E). Subsequently, a nomogram predicting the 1-year, 2-year, and 3-year overall survival probabilities was developed (Figure 2F), and the results showed that the AUC values for 1-year, 2-year, and 3-year overall survival probabilities were 0.705, 0.717, and 0.695, respectively (Figure S3).

### 2.3. Network Establishment for GRB7-Correlated Genes in OC

**Figure 3:**
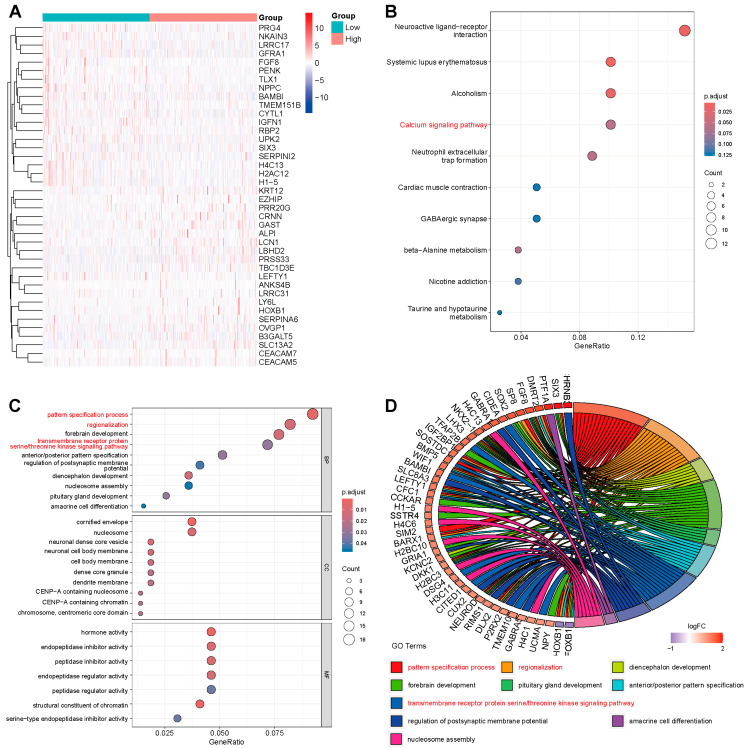
Gene expression and enrichment of GRB7-associated gene in OC from TCGA. (**A**) Heatmap of top 20 genes positively correlated with GRB7 and top 20 negatively correlated genes in OC. (**B**) KEGG enrichment results of all 225 different expressed genes. (**C**) Biological processes, cellular components, and molecular functions from GO enrichment results. (**D**) Chord diagrams of biological processes.

We analyzed the genes associated with GRB7 in CCLE and in TCGA. The results showed that GRB7 was positively correlated with HER2 and CDH1 in CCLE proteomics data (Figure S4A,B), and GRB7 was positively correlated with HER2 and HER2_pY1248 in pan-cancer and ovarian cancer (Figure S4C,D). To further investigate the gene networks associated with GRB7 in OC from TCGA, we analyzed differentially expressed genes (DEGs) in high and low groups based on the median GRB7 mRNA levels. The top 20 positively and top 20 negatively correlated genes with GRB7 were shown (Figure 3A). Next, Kyoto Encyclopedia of Genes and Genomes (KEGG) pathway analysis was performed based on DEGs between the GRB7 high and low groups, and the results showed that the DEGs were mainly involved in several key pathways including the calcium signaling pathway (Figure 3B), an important pathway associated with cancer cell proliferation and invasiveness, and the ability of agents to induce cancer cell death. We also performed a gene oncology (GO) enrichment analysis based on these DEGs and found that there were more genes enriched in tumor progression associated pathways, such as the pattern specification process, regionalization, and the transmembrane receptor protein serine/threonine kinase signaling pathway (Figure 3C,D). Furthermore, we conducted a comprehensive analysis of the association between GRB7’s expression and key driver gene mutations and found that the expression levels of GRB7 were different in wild-type (WT) versus mutant (MUT) genotypes of various driver genes, such as TP53 (Figure S5A,B).

### 2.4. GRB7’s Expression Correlates with Immune Infiltration and Immunotherapy Response and Has Potential to Be a Therapeutic Target

**Figure 4:**
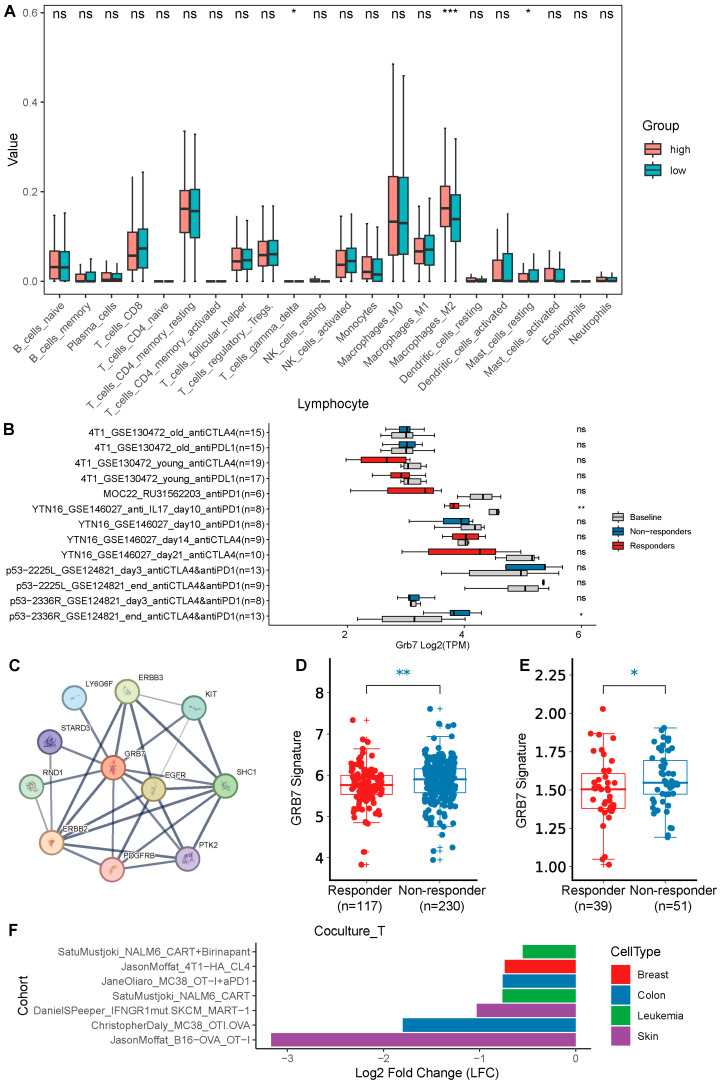
The immune infiltration and association with immunotherapy response of GRB7 in OC. (**A**) Immune cell enrichment in low and high expression levels of GRB7 in OC from CIBERSORT. (**B**) GRB7’s expression levels in responders and non-responders of ICB in syngeneic mouse models. (**C**) Protein–protein interaction signature of GRB7 from string database. (**D**,**E**) The GRB7 signature expression level in responders and non-responders of ICB-treated clinical cohorts, PD1 + CTLA4 in melanoma (**D**), and PDL1 in metastatic urothelial cancer (**E**). (**F**) GRB7 knockout in cancer cells cocultured with T cells from several CRISPR-Cas9 screens. Box plots indicate median (middle line), 25th and 75th percentile (box), and 5th and 95th percentile (whiskers) (**A**,**B**,**D**,**E**), and each dot in the scatter represents an individual patient sample (**D**,**E**). * *p* < 0.05; ** *p* < 0.01; *** *p* < 0.001 by unpaired Student’s *t* test (**A**,**B**,**D**,**E**). ns, not significant.

**Figure 5:**
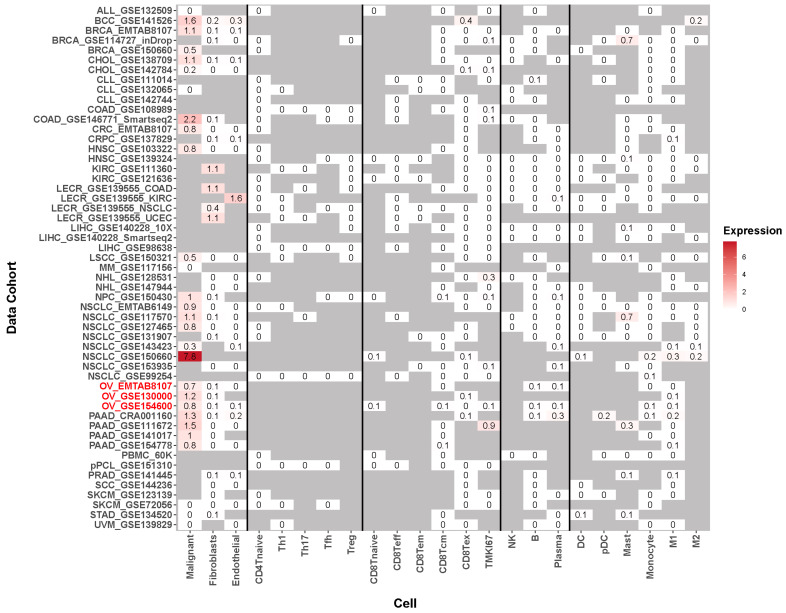
GRB7’s expression among different cell types and datasets. The cohorts highlighted in red are the ovarian cancer single-cell datasets.

To assess the immune infiltration of GRB7 in OC, we first evaluated the enrichment of 20 immune cells with high and low expression of GRB7. The results illustrated that the expression of GRB7 was positively related to M2 macrophages (Figure 4A), which was accepted to be immunosuppressive. Given its immunosuppressive potential, we collected syngeneic mouse models of immune checkpoint blockade (ICB) studies and clinical ICB-treated cohorts and analyzed the expression level of GRB7 between ICB responders and non-responders to evaluate whether GRB7 is associated with immunotherapy response. In these studies, complete or partial response to treatment was defined as responder, and stable or progressive disease was defined as non-response. We found that the GRB7’s expression level tended to be lower in responders than non-responders from syngeneic mouse models and vice versa (Figure 4B). Additionally, we established GRB7 signature, which was calculated by the average expression of genes closely related to GRB7 according to STRING database (Figure 4C) and found that the GRB7 signature tended to be lower in responders than non-responders across two clinical immune checkpoint blockade (ICB)-treated cohorts (Figure 4D,E). Furthermore, we collected various Clustered Regularly Interspaced Short Palindromic Repeats (CRISPR)-associated protein 9 (CRISPR-Cas9) screen datasets [9,10,11,12,13], in which the cancer cells were cocultured with T cells after being edited with CRISPR, and negative log2 fold change in cell viability suggests that knocking out certain genes may sensitize the tumor to T cell killing. These data showed that the deletion of GRB7 in tumor cells, including breast, colon, leukemia, and skin cancers, resulted in heightened susceptibility of the tumor cells to T cell-mediated killing (Figure 4F). Given that GRB7 may affect immunotherapy, we desired to explore whether it could be a good therapeutic target. Targeted therapies aim to interfere with specific cells without affecting others as much as possible, thereby reducing side effects. Therefore, genes that are specifically highly expressed in tumor cells may have potential for targeted therapy. In our quest to determine the therapeutic potential of GRB7, we aggregated multiple single-cell RNA sequencing (scRNA-seq) datasets from diverse studies. The analysis of these datasets revealed a predominant expression of GRB7 in malignant cells as opposed to immune cells, a pattern consistent across not only ovarian cancers but also a broad spectrum of other cancer types (Figure 5).

### 2.5. GRB7 Knockout Inhibits OC Cell Proliferation and Migration

**Figure 6:**
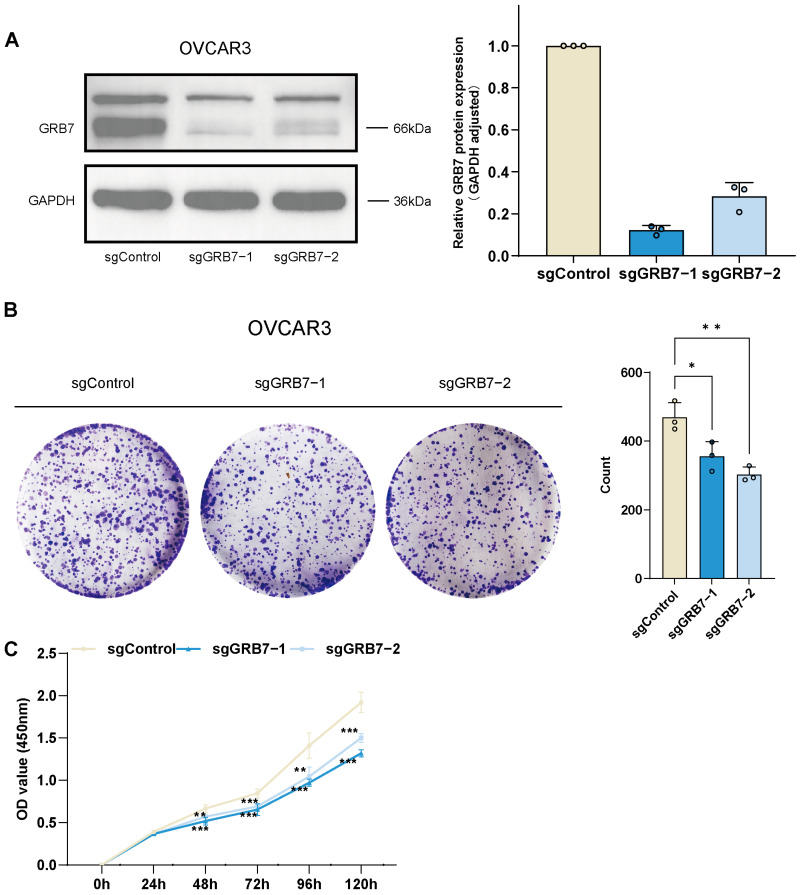
Knockout GRB7 inhibits the proliferation of OVCAR3. (**A**) Western blot analysis of GRB7 knockout efficiency. (**B**) Colony formation capacity of GRB7 knockout and control. (**C**) CCK-8 assay of GRB7 knockout and control. Data are represented as mean ± standard deviation (SD) (**A**–**C**). The Shapiro–Wilk test confirmed normality, and Brown–Forsythe test confirmed homogeneity of variance (**B**,**C**). * *p* < 0.05; ** *p* < 0.01; *** *p* < 0.001 by one-way ANOVA (**B**) and two-way ANOVA (**C**). Data are representative of three independent experiments (**B**,**C**).

**Figure 7:**
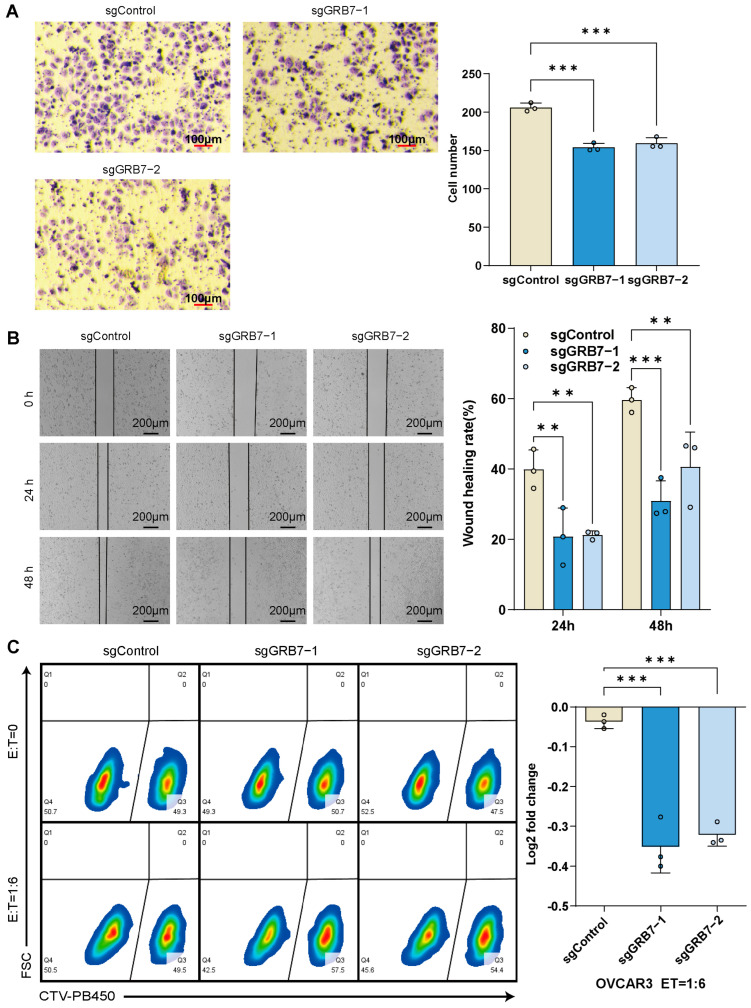
GRB7 knockout in OVCAR3 inhibits cell migration and sensitizes killing effect of CD8+ T cells. (**A**) GRB7 knockout in OVCAR3 reduces migrating cell numbers in transwell assay. (**B**) GRB7 knockout in OVCAR3 slows wound healings. (**C**) The representative FACS results and summary of the log2 fold change in the ratio of GRB7 KO cells over the control after adding CD8+ T cells. The pseudocolor in the figure represents the variation in cell density. Colors range from red to blue, indicating a gradual decrease in cell density from high to low. Data are represented as mean ± SD (**A**–**C**). The Shapiro–Wilk test confirmed normality, and Brown–Forsythe test confirmed homogeneity of variance (**A**–**C**). ** *p* < 0.01; *** *p* < 0.001 by one-way ANOVA (**A**–**C**). Data are representative of three independent experiments (**A**–**C**).

To investigate the functional effect of GRB7 on ovarian cancer, we knocked out GRB7 in the ovarian cancer cell line OVCAR3 and conducted assays to assess proliferation and migration. The knockout efficiency of GRB7 was confirmed by Western blot analyses (Figure 6A). The colony formation and CCK-8 assays results showed that the proliferation of ovarian cancer cells was significantly reduced upon GRB7 knockout (Figure 6B,C). The results of the transwell and wound healing assays showed that GRB7 knockout reduced the migration of ovarian cancer cells (Figure 7A,B).

### 2.6. Enhanced Susceptibility of OC Cells to T Cell-Mediated Cytotoxicity Post-GRB7 Knockout

Our computational analyses implicated that GRB7 knockout in cancer cells would enhance their susceptibility to T cell-mediated cytotoxicity. Therefore, we embarked on a coculture experiment involving tumor cells and T cells. Our results showed that the ablation of GRB7 in the OVCAR3 cell line significantly enhanced the vulnerability of these tumor cells to the cytotoxic effects of CD8+ T cells (Figure 7C).

## 3. Discussion

The GRB7 family of adaptor molecules, which consists of GRB7, GRB10, and GRB14, interact with receptor tyrosine kinases like EGF, HER2, and insulin receptors to play significant roles in cellular signaling [14,15,16]. GRB7 has been identified as a biomarker and found to be important in regulating the cellular signaling pathways involved in proliferation, tumorigenesis, and metastasis. It is implicated in various malignancies, including breast cancer, colon cancer, bladder cancer, and thyroid cancer [7,8,17,18]. Additionally, GRB7 shows promise as a potential therapeutic target in breast cancer as well [19]. According to recent reports, GRB7 was overexpressed in OC and may regulate angiogenesis through the VEGFA/VEGFR2 signaling pathway [20,21]. However, the scope of these findings of OC, derived from a limited number of cell lines and small tumor tissue samples, calls for a more comprehensive analysis of GRB7’s expression and its impact on OC. To address this gap, we conduct rigorous validation through in vitro experiments and use substantial datasets from TCGA, GTEx, CCLE, and GEO, allowing for a comprehensive examination of GRB7 expression patterns in ovarian cancer and their correlation with prognosis.

The overexpression of GRB7 or the co-overexpression of GRB7 and members of the ERBB family play essential roles in advanced human cancers and are associated with decreased survival and recurrence of cancers [6]. In breast cancer, GRB7 was overexpressed and co-amplified with HER2, which promoted cell migration, invasion, and tumorigenesis [8,22]. Additionally, a shorter breast cancer-free interval has been linked to GRB7 overexpression [23]. Moreover, co-treatment of the HER2 inhibitor Herceptin and GRB7 inhibitor in breast cancer resulted in a decrease in the Herceptin EC 50 value [19]. Recently, it was proved that GRB7 was stabilized by circCDYL2 through preventing its ubiquitination degradation and enhanced its interaction with FAK, which thus sustained the activities of downstream AKT and ERK1/2 and contributed to trastuzumab resistance in HER2+ breast cancer patients [24]. HER2 and GRB7 were the two genes most observed in fusion events that were most frequently observed in glioblastoma, breast cancer, and ovarian cancer [25,26]. Additionally, EGF-induced GRB7 tyrosine phosphorylation activates Ras-GTPases and extracellular signal-regulated kinases 1/2 (ERK1/2) contributes to cancer proliferation [27]. Moreover, it was reported that specific GRB7 peptides targeting the SH2 domain of GRB7 blocks EGF/EGFR signal-mediated ERK activation [8] and deletion of GRB7 ablates MMP-9 expression in cervical cancer [28], which suggest that GRB7 may modulate cancer invasion by mediating EGF/EGFR signal-mediated ERK activation or matrix metallopeptidase 9 (MMP-9) expression. In colon cancer, GRB7-PLK1 was also discovered to be a pivotal axis mediating tolerance to MEK inhibitor tolerance [7]. GRB7 also modulated the proliferation, cell cycle, migration and invasion of bladder and thyroid cancer via the AKT pathway and GRB7/ERK/FOXM1 signaling cascade [17,18]. MiR-193a-3p has been reported to target the GRB7 and MAPK/ERK pathways in ovarian cancer, hence promoting the aggressiveness of the malignancy [14]. This work demonstrated that GRB7 is overexpressed in ovarian cancer tissue based on omics data and its overexpression is linked to poor patient outcomes based on TCGA data, consistent with earlier studies on other types of malignancies [8]. From the CCLE and TCGA datasets, it was discovered that GRB7 and HER2 are co-amplified in OC, which is congruent with that in breast cancer [22]. Via TCGA data, we found that the calcium signaling pathway and transmembrane receptor protein serine/threonine kinase signaling pathway were enriched from GRB7 associated genes, echoing the in vitro evidence of reduced proliferation and migration upon GRB7 knockout in OC cells, which involve in cancer progression and resistance to cell death inducers [29,30]. Furthermore, it appears that GRB7 may play a crucial role in carcinogenic pathways due to its variable expression in response to mutations in driver genes like TP53. High-grade serous ovarian cancer is characterized by TP53 mutations in almost all tumors [31]. GRB7 may therefore be particularly important in TP53-mutated OC. Indeed, the data indicate that GRB7 could impact ovarian cancer development via a variety of signaling channels. The possible involvement of GRB7 in vital processes like cell migration and proliferation is highlighted by this interaction.

Although immunotherapy has advanced significantly in the treatment of many cancers, its effectiveness in the management of ovarian cancer is still restricted, with objective response rates under 10% [5,32]. The Ovarian Tumor Tissue Analysis Consortium [33] has demonstrated a clear dose–response relationship between CD8+ tumor-infiltrating lymphocytes (TILs) and improved survival rates in ovarian cancer patients. This underscores the potential of enhancing the effectiveness of immunotherapy in ovarian cancer by further investigating strategies to mobilize pre-existing immunity and boost the activation of T cells within the tumor’s microenvironment [34,35]. The association of GRB7 and HER2 with Type 2 T-helper cells [36], and the role of local Th2 inflammation in fostering an immunosuppressive environment that promotes tumor progression [37,38], has been noted. Our research revealed that the positive association of GRB7’s expression with M2 macrophages infiltration, which may involve immunosuppression [39]. Furthermore, we found that in syngeneic mice or clinical ICB-treated cohorts, the expression level of GRB7 or GRB7 signature tended to be lower in ICB responders than non-responders. The aforementioned details shed light on GRB7’s putative function in the tumor microenvironment and how it affects immune evasion. It’s interesting to note that GRB7-knockout cells have demonstrated enhanced vulnerability to T cell-mediated cytotoxicity, suggesting that targeting GRB7 may improve immunotherapies’ efficacy. This suggests that GRB7 may be a viable target for immunomodulatory strategies targeted at enhancing the effectiveness of immunotherapy in ovarian cancer patients, in addition to serving as a possible prognostic biomarker for the disease.

Molecularly targeted therapies are becoming increasingly attractive due to their specificity for cancer cells while preserving normal cells [40]. In addition to helping to understand the clonal composition and heterogeneity within tumors and identifying the characteristics of tumor cells and immune cells, single-cell sequencing technology offers previously unheard-of opportunities for molecularly targeted therapies, which can provide a crucial basis for identifying new therapeutic targets and drug resistance mechanisms [41,42]. GRB7 may be a promising therapeutic target for ovarian cancer, since our scRNA-seq data analysis showed that it is primarily expressed in malignant ovarian cells. Furthermore, based on TCGA data, we discovered that GRB7 is overexpressed in the majority of tumor tissues and is primarily expressed in malignant cells across a range of cancer types. These results imply that GRB7 may possibly be a potential therapeutic target in multiple tumors.

In conclusion, the data suggest that GRB7 has a great deal of potential as a target for improving the effectiveness of immunotherapy for ovarian cancer. Despite the fact that our study offers compelling evidence, it has certain shortcomings. Large-scale clinical validation studies are needed to assess the expression levels of GRB7 in ovarian cancer patient samples and the association of GRB7 expression with clinicopathological parameters such as tumor stage, grade, even if our systematic analysis of multiomics data has helped us understand the expression pattern of GRB7. Subsequent research endeavors ought to concentrate on elucidating the molecular mechanisms underlying GRB7’s role in ovarian cancer progression and immune evasion, explore how GRB7 interacts with key signaling pathways involved in tumor growth, invasion, and immune modulation within the tumor microenvironment. Additionally, further research is necessary to validate the predictive value of GRB7 and examine the therapeutic benefit of GRB7 targeting in pre-clinical models of OC.

## 4. Materials and Methods

### 4.1. Expression of GRB7 and Clinicopathological Character Analysis

We obtained GRB7’s expression data across various cancers and normal tissues from TCGA [43] (https://portal.gdc.cancer.gov) (accessed on 16 February 2024) and GTEx [44] (https://www.gtexportal.org/home/-index.html) (accessed on 16 February 2024) databases, including clinical samples of ovarian cancer patients from TCGA. A total of 419 OC samples, which included detailed pathology and prognosis information, and 88 normal ovarian tissue RNA sequencing samples from the GTEx database were analyzed. Additionally, GRB7’s expression profiles were obtained from the GEO database (https://www.ncbi.nlm.nih.gov/gds) (accessed on 16 February 2024), and GRB7 protein abundance data were obtained from the cProSite [45], in which the Tandem Mass Tagging (TMT) log2 ratio was used to present the relative protein abundance (https://cprosite.ccr.cancer.gov/) (accessed on 16 February 2024). Immunohistochemistry data of GRB7 protein in human ovarian cancer and normal tissue were retrieved from The Human Protein Atlas [46] (https://www.proteinatlas.org/) (accessed on 16 February 2024).

### 4.2. Correlation Analysis of GRB7 and Prognosis

Patients from TCGA were categorized into two groups according to the median GRB7 mRNA expression level. A Kaplan–Meier survival analysis was performed using the R package survival (v 3.5-8) and survminer (v0.4.9) to assess correlations between GRB7’s expression and OS, PFI, DSS, and DFI of patients with ovarian cancer.

### 4.3. Analyses of Univariate and Multivariate Cox Regression

Univariate and multivariate analyses were conducted with Cox proportional hazards regression models using the R package survival (v3.5-8). A univariate Cox regression analysis and multivariate analysis were used to construct a prognostic classifier to determine the impact of GRB7’s expression levels on the clinical outcomes of OC patients. A nomogram was also constructed incorporating race, stage, age, and GRB7’s expression to predict OS probability. ROC curves were applied to assess the multivariate model’s predictive ability.

### 4.4. Correlation of Related Genes and Gene Set Enrichment Analysis

The differential expression genes were analyzed using DESeq2 (v1.40.2). GO and KEGG enrichment analyses were performed with clusterProfiler (v4.8.3). All visualizations were performed using ggplot2 (v3.5.0), enrichplot (v1.20.3), and GOplot (v1.0.2).

### 4.5. Immune Cell Infiltration and Association with Immunotherapy

The association between immune cell infiltration and GRB7’s expression was evaluated using CIBERSORT algorithm [47]. Additionally, RNA-seq samples of syngeneic mouse tumors treated with ICB in vivo were obtained from the TISMO Database [48] (https://tismo.cistrome.org) (accessed on 8 March 2024) and were used to analyze the association between immunotherapy response and GRB7’s expression. The protein–protein interaction signature of GRB7 was mined from the STRING database [49] (https://string-db.org) (accessed on 8 March 2024), and we compared the signature expression level between responders and non-responders from clinical ICB-treated cohorts [50]. Furthermore, we explored the association of GRB7 knockout with the immune cell killing effect from several CRISPR screen datasets.

### 4.6. GRB7’s Expression Level in Single Cells of Tumor Tissue

We collected various scRNA-seq datasets from the GEO database, including ovarian cancer, non-small cell lung cancer, and breast cancer. We filtered low-quality cells or genes, corrected for batch effects, and normalized all the data. We identified different cell types with their markers and calculated the expression level of GRB7 in the different cell types.

### 4.7. Cell Culture

The human ovarian cancer cell line (OVCAR3) and human embryonic kidney cell line (293T) were obtained from the Cell Bank of the Chinese Academy of Sciences (Shanghai, China). We follow the CRISPR-Cas9 genome engineering protocol to knockout the target gene [51]. Sequences of sgRNAs targeting GRB7 included GCCTTGAGCGACGAGACCTG and GAAGCGGCTATCTCCGCCCA. Following transfection, the cells were selected using puromycin. We validated the knockout efficiency through Western blot analysis, stained with anti-GRB7 (Proteintech, Wuhan, China) and performed relative quantification of GRB7 protein levels based on results from three technical replicates. 293T cells were cultured in Dulbecco’s Modified Eagle’s Medium (DMEM; Gibco, Rockville, MD, USA) supplemented with 10% fetal bovine serum (FBS; Gibco, Rockville, MD, USA) and 1% penicillin and streptomycin (PS; Gibco, Rockville, MD, USA) for lentivirus production for gene editing. RPMI-1640 medium supplemented with 20% FBS (Gibco, Rockville, MD, USA), 1% PS, and 0.01 mg/mL bovine insulin (Coolaber, Beijing, China) was used to culture OVCAR3 cells. The cell lines were cultured in a 37 °C, 5% CO_2_ incubator.

### 4.8. Cell Proliferation

We conducted a CCK-8 assay and colony formation assay to assess cell proliferation. For the CCK-8 assay, 10 μL CCK-8 solution (Dojindo, Ube City, Japan) was added and incubated for 2 h after culturing OVCAR3 cells for 24, 48, 72, 96, and 120 h. The absorbance was then measured at 450 nm using a microplate reader (Sunrise; Tecan, Männedorf, Switzerland). For the colony formation assay, the cells were seeded into 6-well plates at a density of 10,000 cells/well and cultured at 37 °C in a humidified 5% CO_2_ incubator for 10–14 days. The cells were fixed with methanol (Solarbio, Beijing, China) and stained with 0.1% crystal violet (Solarbio, Beijing, China) for 10 min. The number of colonies was counted using ImageJ software (1.54f). All the experiments were performed in three technical replicates.

### 4.9. Cell Migration

We evaluated cell migration using transwell and wound healing assays. For the transwell assay, cells were cultured in an upper chamber (Becton, Dickinson and Company, Franklin Lakes, NJ, USA) with 200 μL of medium containing 2% FBS, while the lower chamber was filled with 500 μL of medium containing 20% FBS. After a 48 h incubation, cells were fixed with methanol and stained with 0.1% crystal violet for 10 min; the experiment was performed in three technical replicates and images from three random fields of each replicate were then acquired via a microscope and counted using ImageJ software, and the average number of cells in the three fields was used for a quantitative analysis. For the wound healing assay, after confirming the ideal cell density under a microscope, a pipette tip was used to create a wound scratch; then, the cells were cultured for 48 h. Images were taken at 0, 24, and 48 h; the experiment was performed in three technical replicates.

### 4.10. In Vitro Cancer-Killing Assay by Antigen-Specific T Cells

Primary CD8+ T cells were isolated from a donor’s PBMC (Biosource, Waltham, MA, USA) following the protocols of the CD8+ T isolation kit (Stemcell, Vancouver, BC, Canada); then, we overexpressed 1G4 TCR, specific to the tumor antigen NY-ESO-1, in primary CD8+ T cells, and OVCAR3 cells were engineered to express NY-ESO-1 simultaneously, as described in a prior study [52]. We pre-plated the Cell Trace Violet (CTV; Invitrogen, Waltham, MA, USA) stained control and GRB7 knockout cells at a 1:1 ratio and cocultured them with 1G4 CD8+ T cells at a 1:6 E:T ratio in triplicates. After 24–48 h of coculturing, cells were collected and analyzed using Cytoflex (Beckman Coulter, Brea, CA, USA) and FlowJo software (v10.8.1). The experiment was performed in three technical replicates.

### 4.11. Statistical Analyses

The data were analyzed using GraphPad Prism (v9.5.1) and R software (v4.3.0). Statistical analyses were performed using parametric tests where appropriate. The normality of the data was assessed using the Shapiro–Wilk test, and the homogeneity of variance was evaluated using the Brown–Forsythe test. The data were considered to follow a normal distribution if the Shapiro–Wilk test *p*-value was greater than 0.05. Homogeneity of variance was confirmed if the Brown–Forsythe test *p*-value was greater than 0.05. If these assumptions were not met, non-parametric tests were used. *p* < 0.05 was considered statistically significant.

## 5. Conclusions

In conclusion, GRB7 emerges from our study as a potential biomarker for ovarian cancer prognosis and a promising target for therapeutic intervention. Our integrative approach spanning transcriptomic and proteomic data, complemented by functional assays, provides a robust framework for understanding GRB7’s multifaceted role in OC. As we progress toward precision oncology, the insights from this research lay the groundwork for identifying therapeutic targets for ovarian cancer.

## Data Availability

The data presented in this study are available in this article and supplementary material.

## Supplementary Materials

The following supporting information can be downloaded at https://www.mdpi.com/article/10.3390/ph17081043/s1, Figure S1: The expression level of GRB7 in different cancer cell lines from CCLE; Figure S2: Quantification of immunochemically stained GRB7 proteins in OC and normal ovarian tissue from Human Protein Atlas; Figure S3: The time-dependent ROC curves of the risk score model for predicting 1-year, 3-year, and 5-year overall survival in OC; Figure S4: Volcano plots depicting the association of GRB7 with other proteins in cancer; Figure S5: Comprehensive analysis of the association between GRB7’s expression and key driver gene mutations.
