# Supplementary material for: GRB7 Plays a Vital Role in Promoting the Progression and Mediating Immune Evasion of Ovarian Cancer
Source: Pharmaceuticals (Basel). 2024 Aug 7;17(8):1043. doi: 10.3390/ph17081043 (PMC11357674; doi:10.3390/ph17081043)
Supplement: Supplementary file 1 [file pharmaceuticals-17-01043-s001.zip › pharmaceuticals-3025575-supplementary.pdf]

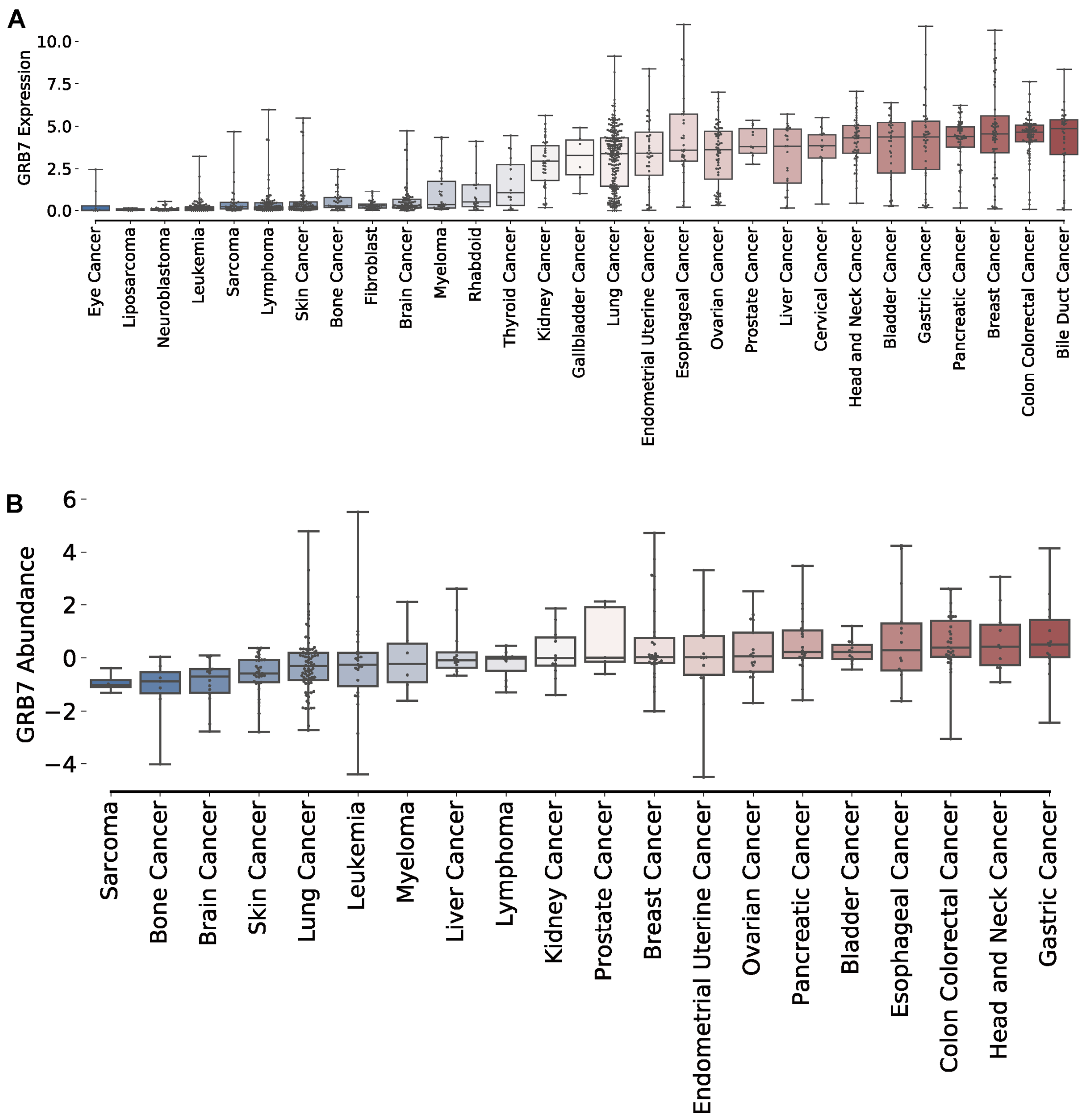

Figure S1. The expression level of GRB7 in different cancer cell lines from CCLE. (A) GRB7 mRNA levels in cancer cell lines. (B) GRB7 protein levels in cancer cell lines.

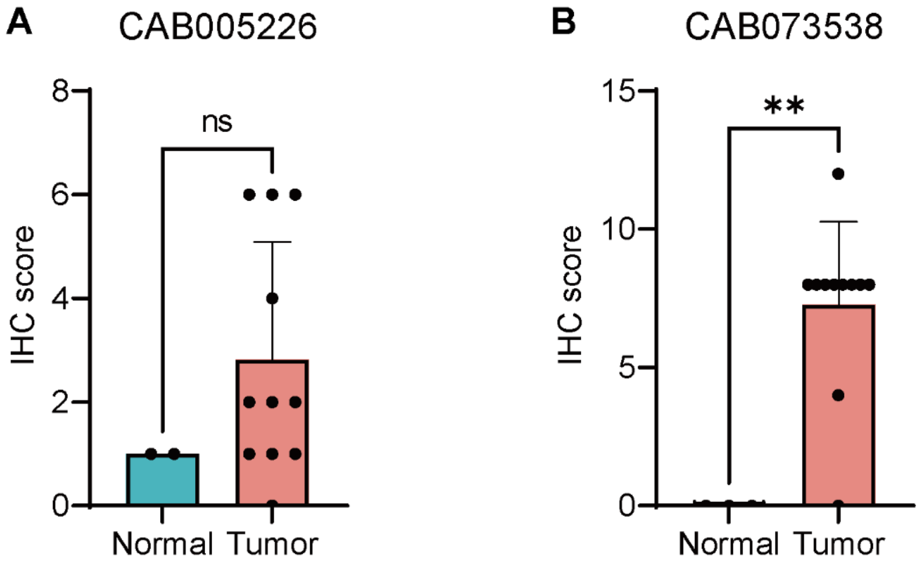

Figure S2. Quantification of immunochemical stained GRB7 proteins in OC and normal ovarian tissue from Human Protein Atlas. (A) Quantification results of GRB7 stained with antibody CAB005226. (B) Quantification results of GRB7 stained with antibody CAB073538. ** $p < 0.01$.

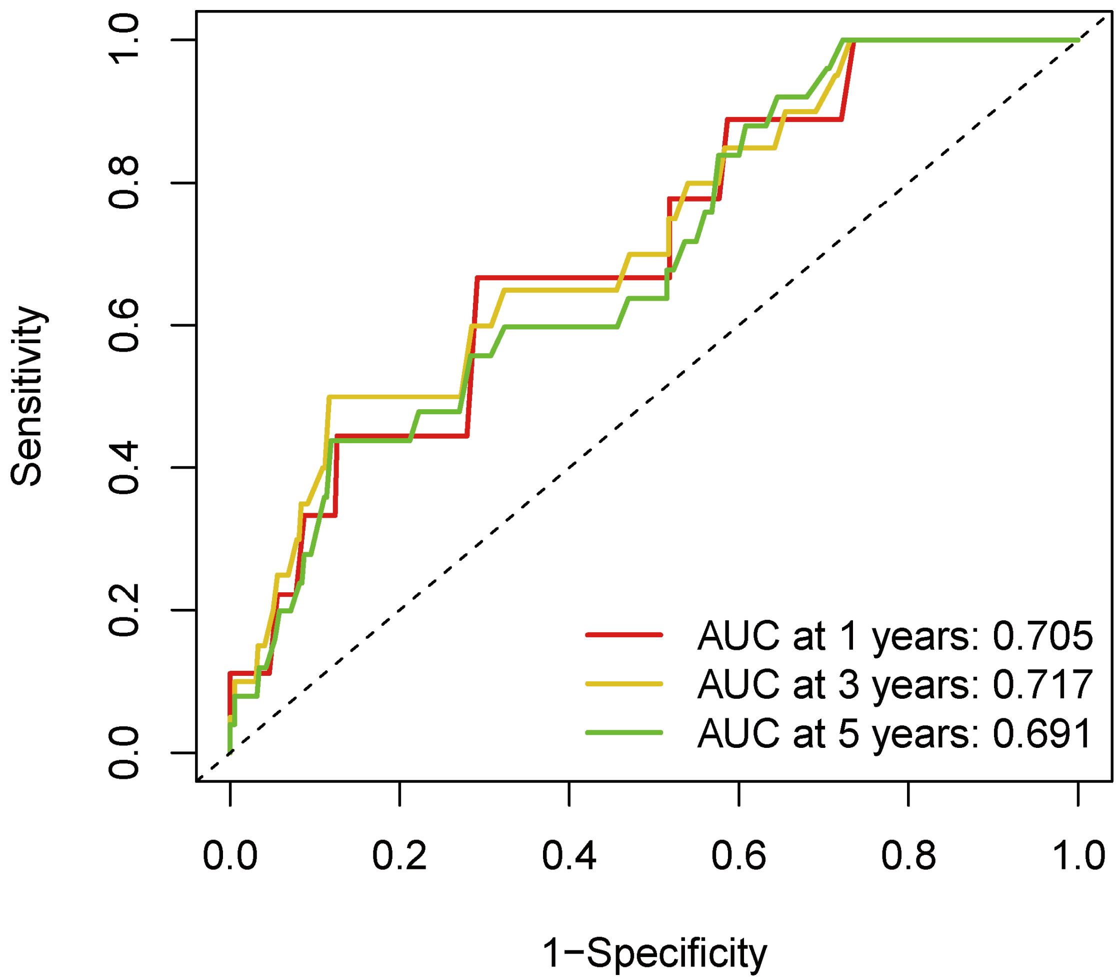

Figure S3. The time-dependent ROC curves of the risk score model for predicting 1-year, 3-year and 5-year overall survival in OC.

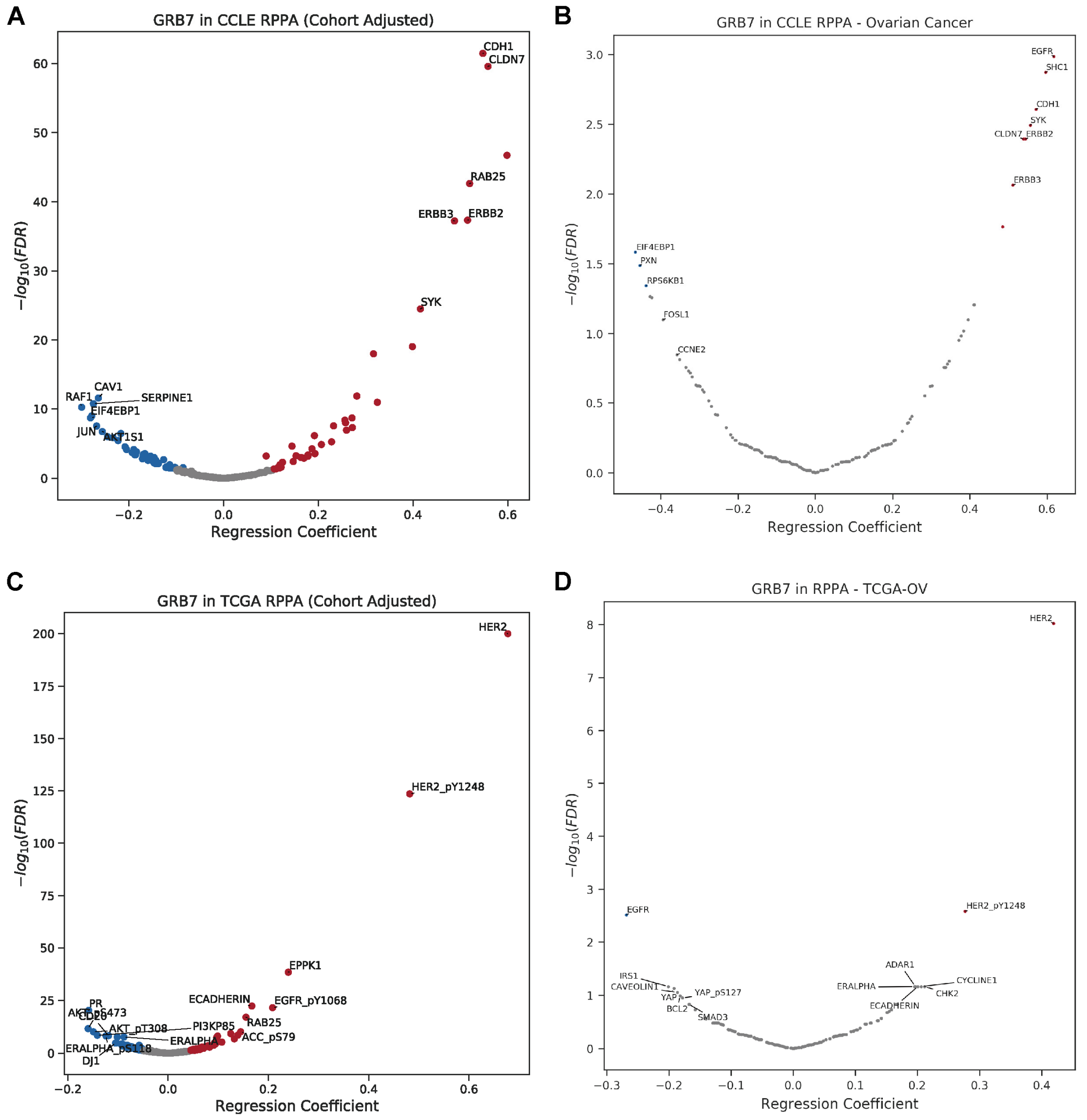

Figure S4. Volcano plots depicting the association of GRB7 with other proteins in cancer. (A-B) Associated proteins with GRB7 of pan-cancer and ovarian cancer in CCLE proteomics. (C-D) Associated proteins with GRB7 of pan-cancer and ovarian cancer in TCGA reverse phase protein array.

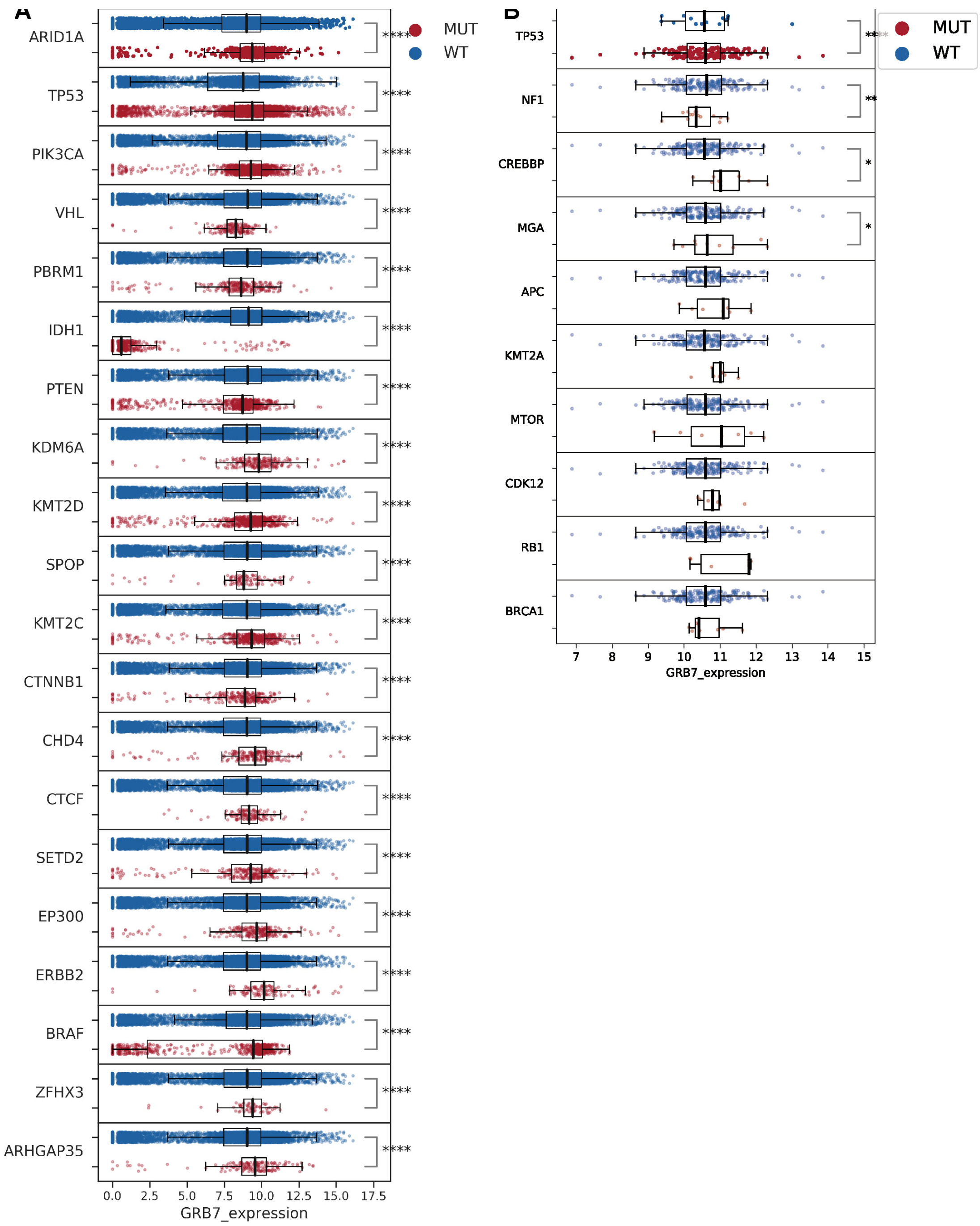

Figure S5. Comprehensive analysis of the association between GRB7 expression and key driver gene mutations across multiple cancer types(A) and specifically in ovarian cancer (B).
